# Obtaining a Wire of Biocompatible Superelastic Alloy Ti–28Nb–5Zr

**DOI:** 10.3390/ma13092187

**Published:** 2020-05-09

**Authors:** Elena O. Nasakina, Sergey V. Konushkin, Maria A. Sudarchikova, Konstantin V. Sergienko, Alexander S. Baikin, Alena M. Tsareva, Mikhail A. Kaplan, Alexey G. Kolmakov, Mikhail A. Sevost’yanov

**Affiliations:** A.A. Baikov Institute of Metallurgy and Material Science RAS (IMET RAS), Institution of Russian Academy of Sciences, Leninsky Prospect, 49, 119991 Moscow, Russia; venev.55@mail.ru (S.V.K.); bloodymaria@list.ru (M.A.S.); shulf@yandex.ru (K.V.S.); baikinas@mail.ru (A.S.B.); alyo.tsaryowa2012@yandex.ru (A.M.T.); mishakaplan@yandex.ru (M.A.K.); imetranlab10@mail.ru (A.G.K.); cmakp@mail.ru (M.A.S.)

**Keywords:** shape memory alloy, titanium, superelasticity, biocompatibility, niobium, zirconium

## Abstract

Using the methods of electric arc melting, intermediate heat treatments, and consecutive intensive plastic deformation, a Ti–Nb–Zr alloy wire with a diameter of 1200 μm was obtained with a homogeneous chemical and phase (β-Ti body-centered crystal lattice) composition corresponding to the presence of superelasticity and shape memory effect, corrosion resistance and biocompatibility. Perhaps the wire structure is represented by grains with a nanoscale diameter. For the wire obtained after stabilizing annealing, the proof strength Rp0.2 is 635 MPa, tensile strength is 840 MPa and Young’s modulus is 22 GPa, relative elongation is 6.76%. No toxicity was detected. The resulting wire is considered to be promising for medical use.

## 1. Introduction

Medical materials for the manufacture of implants should exhibit biomechanical compatibility (superelasticity, low Young’s modulus, delayed response to influence), corrosion resistance, and biological inertness.

The behavior close to the behavior of living tissues is demonstrated by shape memory alloys, especially alloys of the Ti–Ni system [1,2,3,4,5,6,7]. Since their discovery in the 1960s, there have been detailed studies of their properties such as martensitic transformations, delay effect, hysteresis phenomena, shape memory effect, superelasticity, damping properties, features of obtaining porous preforms and composite materials based on Ti–Ni, corrosion properties in static and dynamic conditions, electrochemical behavior, toxicology and carcinogenic properties, properties after thermal cycling and its effect on changes in the temperature of the beginning and end of austenitic and martensitic transformations in alloys [1,2,3,4]. This has led to their widespread use in the automotive, aerospace, and industrial fields as compact, silent and very lightweight drives and sensors, and to the prospect of replacing conventional executive systems with these materials [5]. But the main thing is that Ti–Ni–based alloys are widely used in medicine, including in such areas as dentistry, orthopedics, and vascular and neurological surgery [6]. Among the features of Ti–Ni for medical use, in addition to the shape memory effect and superelasticity, other properties are also noted: elastic deployment, thermal deployment, kink resistance, constant stress, dynamic interference, stress hysteresis (biased stiffness), temperature dependence of stress. These properties allow the creation of special medical devices that facilitate operations: hingeless nitinol (Ti–Ni) grasper, biopsy forceps, lntraortic balloon pumps with superelastic central lumen [7].

At the same time, it is noted that the properties of the material largely depend on the chemical composition of the surface, which sharply depends on the method of preparation: the surface concentration of Ni can vary between 0–27% [4]. The presence of the outstanding properties of Ti–Ni–based alloys (such as the shape memory effect, superelasticity, high wear resistance, and others) simultaneously causes certain difficulties in its mechanical processing by traditional methods, which forces scientists to study other methods of its treatment [8,9].

Moreover, the toxic properties of nickel and the likelihood of corrosion failure of the material (damage to the product in the environment of operation) limit their applicability [10,11,12,13,14,15,16,17]. The inhibitory effect of nickel ions on cell growth and survival [10,15] and the inflammatory effect of the nickel implant on surrounding tissues [14] have been shown. The degree of tissue damage was closely related to the concentration of dissolved Ni. In [13], it was noted that a wire made of pure nickel caused severe inflammation at a distance of 5 mm from the implantation site in rats with necrosis of 1 mm around the implant. Importantly, nickel concentrations, in this case, reached 48 mg/g near the implants, exponentially falling to undetectable levels at a distance of 3–4 mm from the implants. In [11], it was demonstrated that the corrosive products of the nitinol stent wire were potentially toxic to rat aortic smooth muscle cells.

At the same time, it is potentially possible to obtain shape memory alloys from non-toxic metals [18,19,20,21,22,23,24,25,26,27,28]. The shape memory effect was noted for the Ti–35Nb alloy (wt.%) [18]. It was shown in [24] that the TiNb alloys with a niobium content of 22–27 at.% exhibit the superelastic behavior and the shape memory effect; however, they contain only non-toxic elements. The superelasticity of TiNb alloys is based on the reversible martensitic transformation of the high-temperature β-phase with the bcc lattice into the low-temperature α”-phase with the rhombic lattice β ↔ α”. The maximum lattice deformation of the initial phase during this transformation is called the crystallographic reversible deformation resource, which is a quantitative characteristic of the superelastic behavior of the material. Doping of titanium with β-isomorphic elements (Nb, Mo, Ta) inhibits diffusion and leads to the formation of the α”-phase by the martensitic mechanism during quenching. The higher their concentration, the lower the temperature of the stable state of the β-phase. For example, it was shown that the superelastic deformation of the Ti–22Nb (at.%) alloy increased with the addition and increase of Ta content [19]. In [23], it was shown that the martensitic transformation β ↔ α″ is also characteristic of Ti–Ta alloys.

In [21], the effect of adding Zr to the Ti–22Nb (at.%) binary alloy on the shape memory effect and mechanical properties was studied. Usually, in titanium alloys, zirconium acts as a neutral hardener, however, in alloys of β-titanium (body-centered cubic lattice), it can also act as a β-stabilizer, lowering the temperature of phase transformation, and suppresses the formation of the non heat-conductive ω-phase. Besides, zirconium has a larger atomic radius (0.160 nm) compared with titanium and niobium (0.145–0.146 nm). Therefore, its introduction into the crystal lattice of the TiNb alloy should contribute to an increase in the interatomic distance, a decrease in the bonding strength between atoms, which means a decrease in the elastic modulus (Young). The shape memory effect and superelasticity were observed in Ti–22Nb alloys with a zirconium content of 2–6 at.%, but were already absent at 8 at.%

In [20], phase transitions and martensitic transformation products of biocompatible quenched Ti–Nb–Ta(Zr) alloys were studied. The existence of two successive mechanisms of reversible deformation accumulation is shown: lattice distortion of rhombic martensite and β ↔ α″ phase transition. A reversible phase transition is manifested in certain Ti–Nb–(Ta, Zr) alloys, characterized by high elementary cell deformation, extremely low moduli of elasticity, and the exception of metastable ω phase formation.

It was found that phase transformations in the alloys of Ti–(13–26)Nb–(22–38)Ta (wt.%) and Ti–(13–35.5)Nb–(5–22)Ta–(4–7.2)Zr (wt.%) are sensitive to both composition and cooling rate [26]. The addition of Zr stabilized the β phase, lowered the initial temperature of martensite transition, and suppressed the formation of the ω phase. In [27], Ti–29Nb–13Ta–4.6Zr alloy (wt.%) is considered as a candidate for biomedical applications. This alloy has a low Young’s modulus close to the bone modulus (10–30 GPa). This is achieved by including one metastable β-phase due to a large number of alloying elements.

Thus, these alloys can be considered as perspective materials for medical manufacture, for example for “stent” and “cava-filter” devices. At the same time, there is no information on the production of these alloys in the form of a thin wire, which is needed for the production of designated medical devices, while the structure and properties of the material change significantly during intense plastic deformation accompanying the sequential transfer of the melted workpieces into wire samples.

Therefore, this work aimed to investigate the possibility of obtaining a wire of Ti–28Nb–5Zr alloy and its operational properties.

## 2. Materials and Methods

Iodide titanium, iodide zirconium, and technically pure niobium were used as charge materials.

Smelts were melted in an electric arc vacuum furnace with a non-consumable tungsten electrode LK8 from LEYBOLD-HERAEUS (Cologne, Germany) at a residual and working (argon) pressure of 1.33 Pa and 2.03 GPa, respectively. The duration of each melting of one ingot weighing 30 g was 1–1.5 min. Before melting the ingot, the getter melted. An ingot of iodide zirconium weighing 15–20 g was used as a getter. The first 2 remelts did not lead to uniform melting of the elements. Thus, the first 3 remelts were intended to produce a single ingot. Further remelting (5 and 7 times) was carried out with the aim of a possible increase in the homogeneity of the composition.

Furthermore, the obtained ingots were fused into a single ingot weighing 180 g for 2 remelting.

Large ingots were additionally subjected to homogenizing annealing in the vacuum of 6.67 MPa at a temperature of 850–1000 °С for 12 h and cooled together with the furnace. An SSHVZ-1.2.5/25-I3 furnace was used (MosZETO, Moscow, Russia).

Rolling took place in the air on reversing mills DUO-300 (Istok ML, Moscow, Russia) to a cross-section of 10 × 10 mm^2^ with a degree of deformation (ratio of the cross-sectional area of the ingot before deformation to the cross-sectional area after deformation) 1.2. The billets were heated in air to 600 °C immediately before deformation.

The rods were annealed in a PTS-2000-40-1200 feed-through tubular electric furnace, manufactured by Lori-Thermo LLC (Podolsk, Russia).

To obtain a wire with a diameter of 1.5 mm (a bar of cylindrical section) from a bar, rotational forging was carried out sequentially on radial forging machines B2129.02, B2127.01, B2123.01 (Pressmash, Taganrog, Russia) with a successive change of strikers with diameters: 13; 12; 11; 10; 9; 8; 7.2; 6.5; 5.8; 5; 4.7; 4.25; 3.8; 3.4; 3; 2.7; 2.45; 2.2; 2.0; 1.6. Billets were heated in the air immediately before deformation in the PTS-2000-40-1200 furnace to 600 °C.

From a wire diameter of 1.5 mm to a diameter of 1.2 mm, drawing was carried out on a C7328/ZF machine by THE NORTHWEST MACHIBE CO.LTD (Xi’an, Shaanxi, China). The drawing took place in the air at a die temperature of 150 °C. Aquadag was used as a lubricant. The drawing speed was 2–6 m/min. The diameter step of the dies was 0.1 mm. Before drawing, annealing was carried out for 10 min at 600 °С in air. At the necessary stages, heating of the wire to 600 °C for 10–20 min, cooling, quenching in aquadag from a temperature of 200–300 °C were used.

The wire was also subjected to heat treatment at a finite diameter to stabilize the structure and properties of the material in a vacuum of 6.67 mPa at a temperature of 500–800 °C for 1 h after ultrasonic cleaning in a washing solution. At the final stage, the samples were polished through felt disks with a suspension with particles of 6, 3, 1 μm.

After obtaining ingots, rods, and wire, we studied the uniformity of the elements distribution of the Ti–Nb–Zr alloy using Auger spectrometry, a structure using optical and scanning electron microscopes. Investigations were made of the structure, composition, and morphology of the surface of the Ti–Nb–Zr wire after drawing and their changes after the surface and bulk heat treatments.

Samples were prepared for metallographic studies by grinding after pressing on a Piatto diamond disk with P120 grit, P320 abrasive paper, P800, on a diamond grinding disk Aka-Allegran-3 with a suspension of DiaMaxx Poly with a diamond size of 6 microns, on Akasel DARAN woven acetate with DiaMaxx Poly suspension with 3 μm diamond sizes, on Akasel NAPAL velvet with a suspension of DiaMaxx Poly with a diamond size of 1 μm, on Akasel CHEMAL foamed neoprene with an Akasel Colloidal Silica suspension with a grain size of 50 nm.

For microstructural analysis, the samples were etched for 5–10 s using a mixture of HF:HNO_3_:H_2_O in a ratio of 1:2:47 mL, washed several times with distilled water and alcohol and then air dried. A metallographic optical microscope (Neophot 2, Carl Zeiss, Oberkochen, Germany) with digital image-processing capability was used.

Scanning electron microscopy (SEM; TESCAN VEGA II SBU, TESCAN, Brno, Czech Republic) with energy-dispersive X-ray spectroscopy (EDS, INCA Energy, Brno, Czech Republic) and Auger electron spectroscopy (JAMP-9500F, JEOL Co., Tokyo, Japan) with accompanying ion beam etching (argon bombardment) at an angle of 30° were also used. All samples were ultrasonically cleaned in alcohol immediately before analysis.

The phase composition of the samples was determined by X-ray diffraction (XRD; Ultima IV, Rigaku Co., Woodlands, TX, USA) using Cu Kα radiation with a graphite monochromator, vertical goniometer, and rapid semiconductor detector (D/teX). The Bragg–Brentano method was employed. Phase analysis was conducted using the PDXL program complex (Rigaku Co., Woodlands, TX, USA) and ICDD database.

The static mechanical properties of samples with a working part length of 45 mm were determined on an Instron 3382 (Instron, MA, USA) universal testing machine according to GOST RF standard 10446-80 (ISO 6892-1:2019(E)) [29]. The base diameter was used in the calculation of strength properties. Three to five samples were tested per one experimental point. The tensile strength Rm, proof strength, plastic extension Rp0.2, percentage elongation after fracture A, and the Young’s modulus Е (modulus of elasticity) were determined.

In the biocompatibility study, SH-SY5Y neuroblastoma cells cultured in DMEM (Gibco) supplemented with 10% bovine serum (FBS, Gibco, Russia) and 15 μg/mL gentamicin were used. The culture was left in a CO_2_ incubator (95% air, 5% CO_2_, humidity 100%) for 72 h. Cells were stained with fluorescent dyes Hoechst 33342 (Hoe, 5 μM), propidium iodide (PI, 3 μM), MitoTracker Deep Red FM (MTDR, 0.3 nM) for 20 min in Hanks solution supplemented with 20 mM HEPES, pH = 7.36, then washed for 10 min in Hanks solution without dyes. The studies were performed using a Leica DMI6000 B inverted fluorescence microscope using a Leica HCX PL APO 63x/1.40-0.60na OIL objective (Leica Microsystems GmbH, Wetzlar, Germany). In each sample, at least 3 regions were randomly selected. The mitotic cell index in the logarithmic growth phase (3 d after inoculation) determined mitotic activity [16,30].

## 3. Results

After smelting, the ingots were visually examined over the cross-section. No explicit undissolved particles of the starting elements were found. Triple remelting of ingots allowed a homogeneous chemical composition to be obtained. A fairly uniform distribution of elements over the volume of ingots was revealed, based on a comparison of alloy compositions in randomly selected areas of analysis, as well as good agreement between the obtained values of metal concentrations in the alloy and the calculated values expected based on selected weights of charge materials.

**Figure 1:**
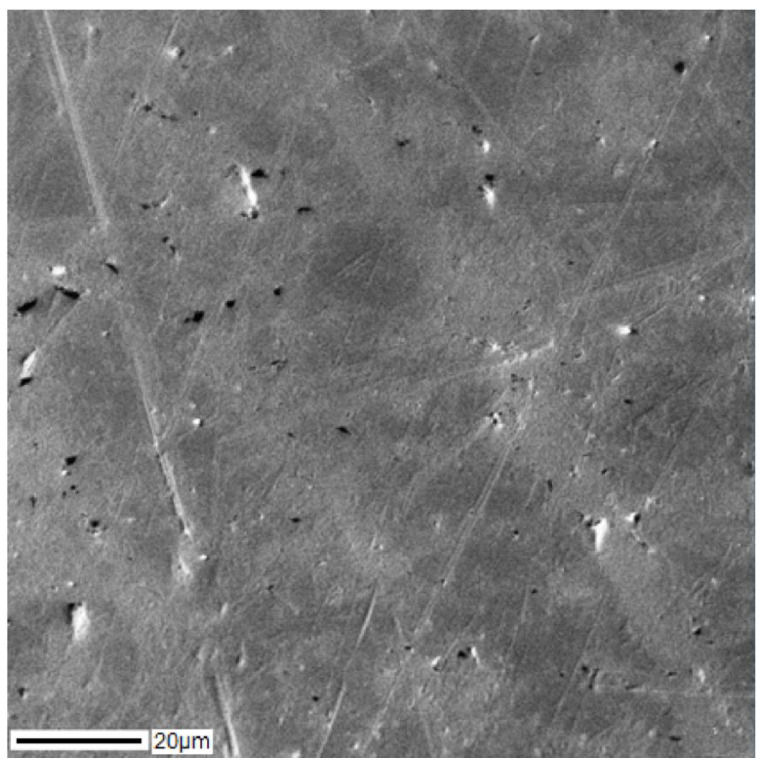
The dendritic structure of the Ti–28Nb–5Zr alloy after seven-fold remelting obtained using scanning electron microscopy (SEM).

**Figure 2:**
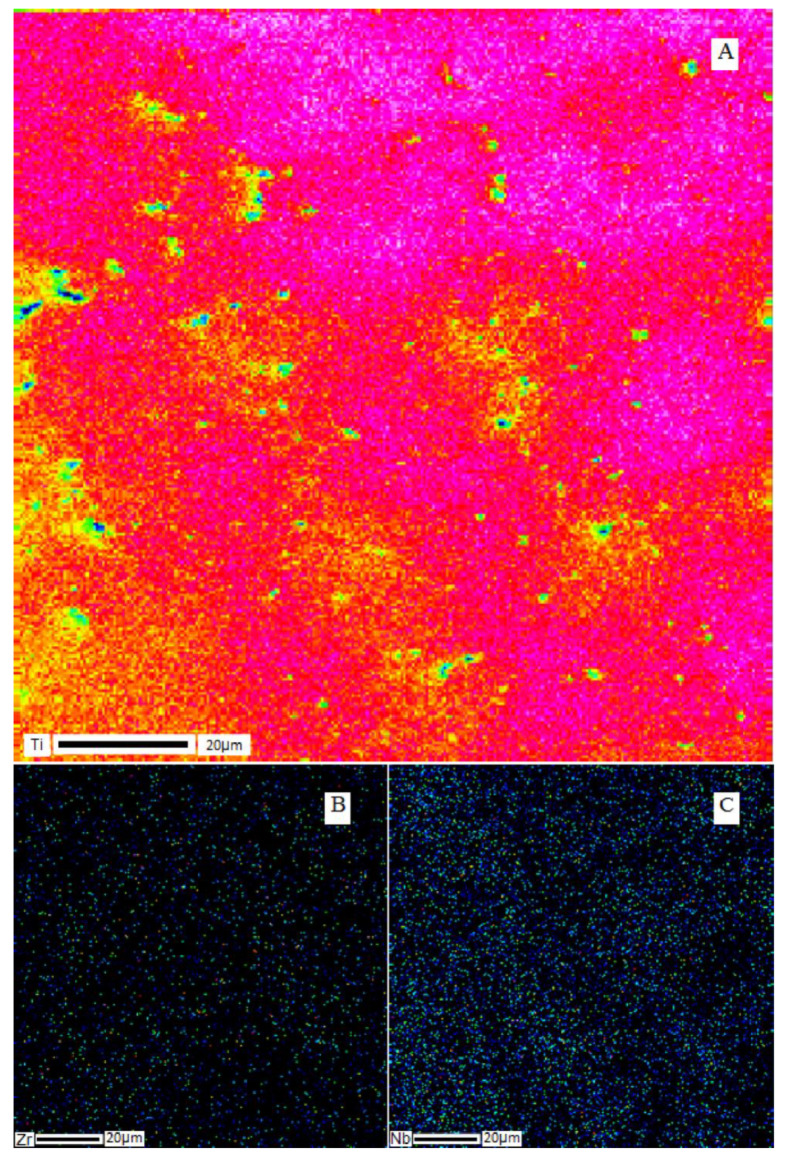
Elemental distribution of metals in the dendritic structure of the Ti–28Nb–5Zr alloy: (**A**) titanium; (**B**) zirconium; (**C**) niobium.

SEM images of the cross-section of the Ti–28Nb–5Zr alloy ingot are shown in Figure 1. Dendritic structures at a high resolution were hardly readable, because of large sizes of branches (axes), but they were easily distinguished during elemental mapping. The results of the analysis of the elements’ distribution in the Ti–28Nb–5Zr alloy in the dendritic structure are presented in Figure 2.

It can be seen that niobium and zirconium were distributed uniformly throughout the volume of the samples, titanium was concentrated in the dendrites themselves, but was also found in the areas between the axes of the dendrite.

The distribution of elements according to Auger spectrometry, as well as X-ray phase analysis, indicates the formation of a single structure and the absence of fragments of individual metals.

**Figure 3:**
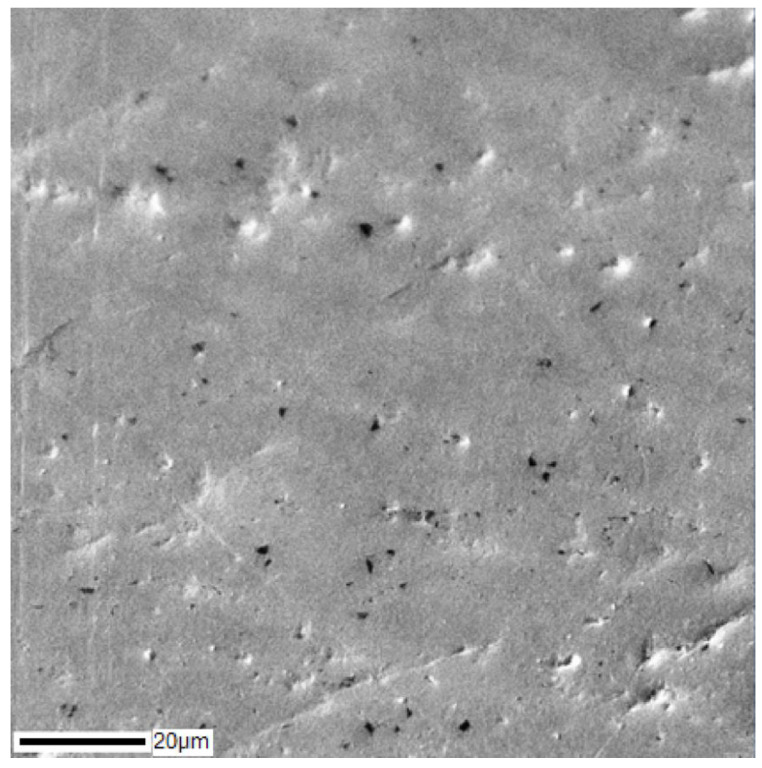
The structure of the Ti–28Nb–5Zr alloy after annealing at 900 °C for 12 h, obtained using SEM.

**Figure 4:**
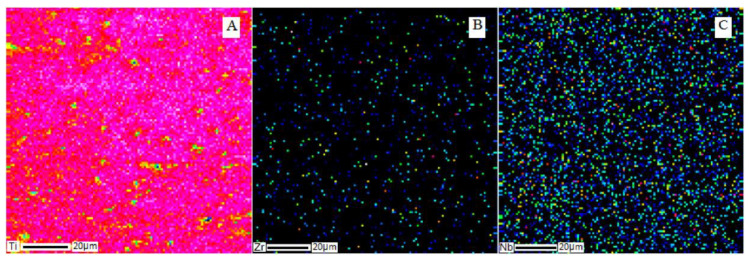
Elemental distribution of metals in the structure of the Ti–28Nb–5Zr alloy after annealing at 900 °C for 12 h: (**A**) titanium; (**B**) zirconium; (**C**) niobium.

To destroy the dendritic structure, annealing was carried out at temperatures of 850–1000 °С. SEM images of the cross-section of the Ti–28Nb–5Zr alloy ingot after annealing at 900 °C for 12 h are shown in Figure 3, and the results of the analysis of the alloy elements’ distribution are shown in Figure 4. Homogeneous distribution of all elements was noted, the dendrites were dissolved.

**Figure 5:**
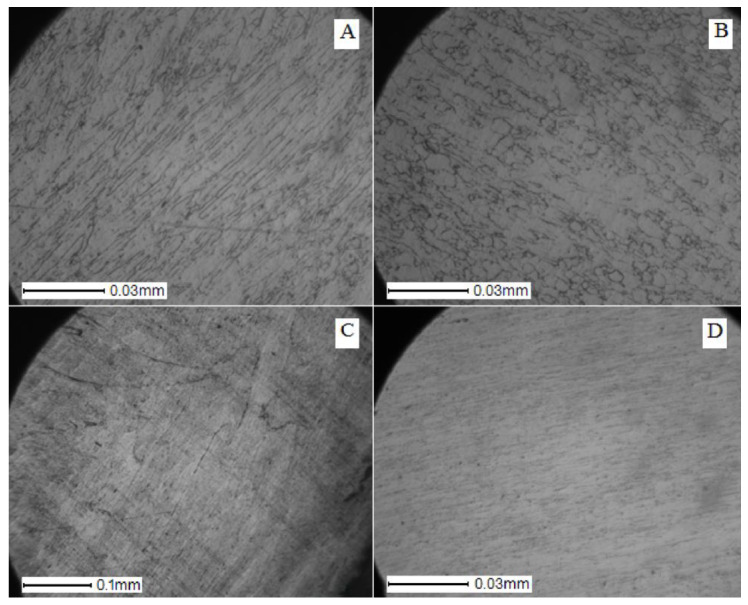
Photos of the microstructure of bars of the Ti–28Nb–5Zr alloy with (**A**) 8, (**B**) 4, (**C**) 2.5, and (**D**) 1.5 mm diameters in the longitudinal direction of the section.

**Figure 6:**
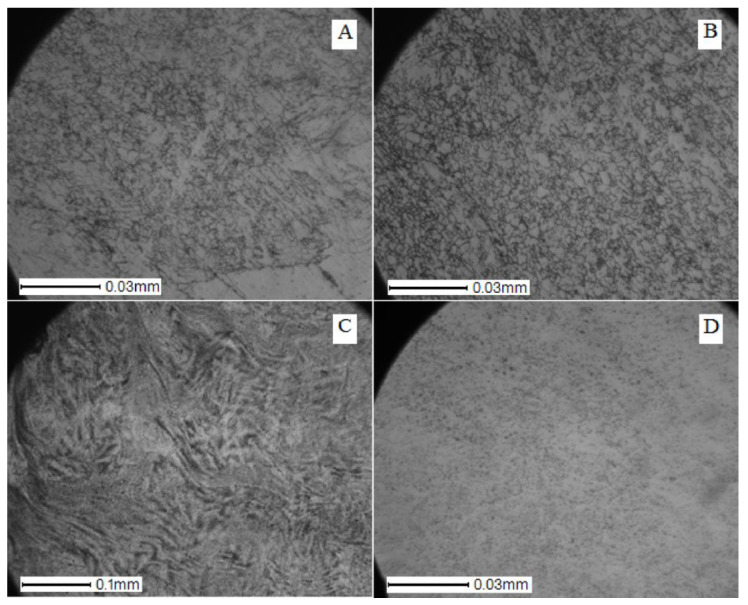
Photos of the microstructure of bars of the Ti–28Nb–5Zr alloy with (**A**) 8, (**B**) 4, (**C**) 2.5, and (**D**) 1.5 mm diameters in the transversal direction of the section.

**Figure 7:**
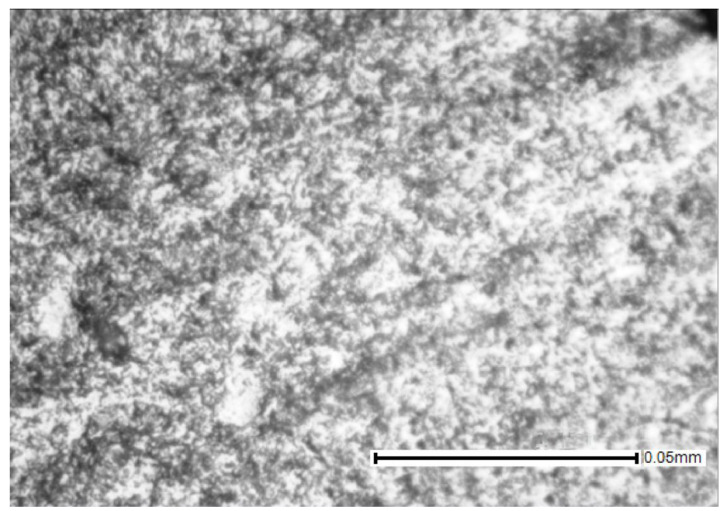
Microstructure of Ti–28Nb–5Zr wire after drawing.

Photographs of the microstructure of the Ti–28Nb–5Zr alloy rods with a diameter of 8, 4, 2.5, and 1.5 mm are shown in Figure 5 and Figure 6. Photographs of the microstructure of the wire after drawing are shown in Figure 7.

The grains in the test samples had a pronounced texture in the direction of rolling and forging. The rods had a layered structure along the longitudinal section and radial in the perpendicular section. On a cross-section of a bar with a diameter of 8 mm, grains had a length of several tens to hundreds of microns, a width of 1 to 10 microns. There were “dark” zones with finer grains (perhaps these zones accounted for the main deformation during forging).

On a longitudinal cross-section of a bar with a diameter of 4 mm, layers became more visible. The grains on the longitudinal section have become shorter; more deformed “dark” areas became more. In the “dark” areas the grains were up to several tens of microns in length. The width of the grains also decreased to a few microns.

The downward trend in grain size continued. In a bar with a diameter of 1.5, the grains became less than 1 μm in width and less than 10 μm in length.

**Figure 8:**
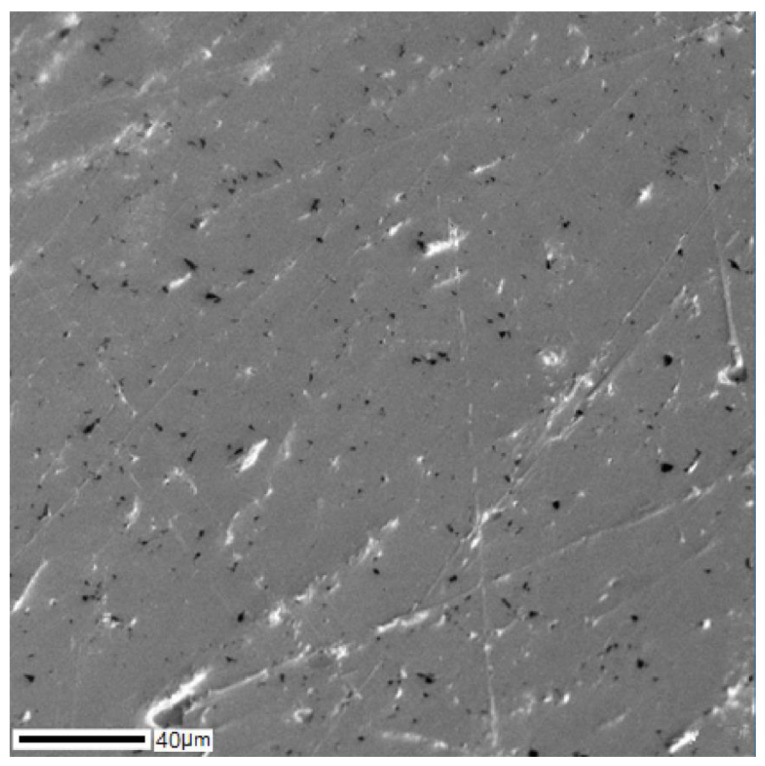
The structure of the Ti–28Nb–5Zr rod obtained using SEM.

**Figure 9:**
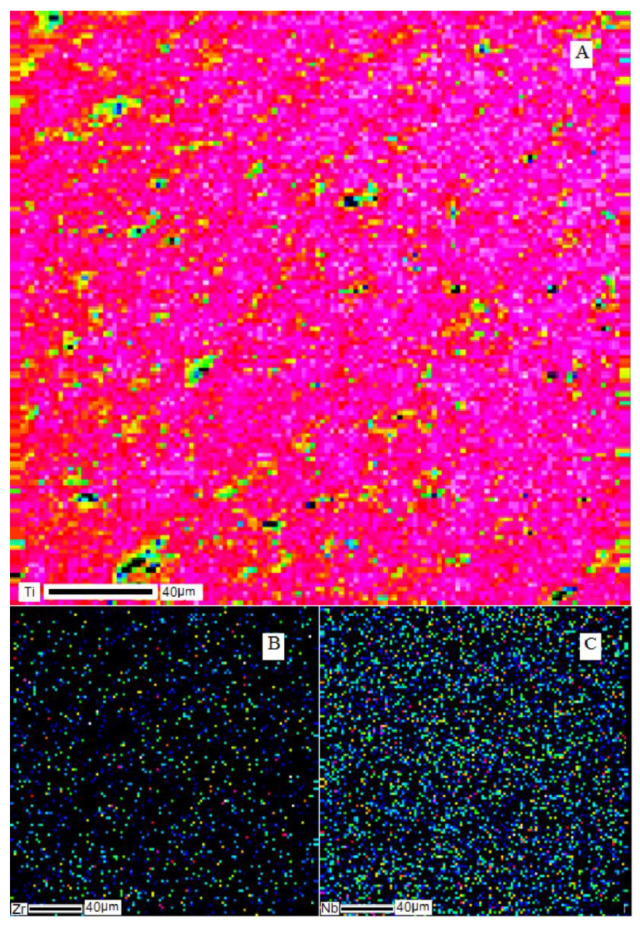
The element-wise distribution of metals in the structure of the Ti–28Nb–5Zr bar: (**A**) titanium; (**B**) zirconium; (**C**) niobium.

The SEM image of the cross-section of the Ti–28Nb–5Zr bar is shown in Figure 8, and the results of the analysis of the distribution of alloy elements are shown in Figure 9. A uniform distribution of all elements was noted. On the map of titanium, heterogeneity was observed, which was due only to the textured surface as a result of polishing, and not to the titanium content gradient.

**Figure 10:**
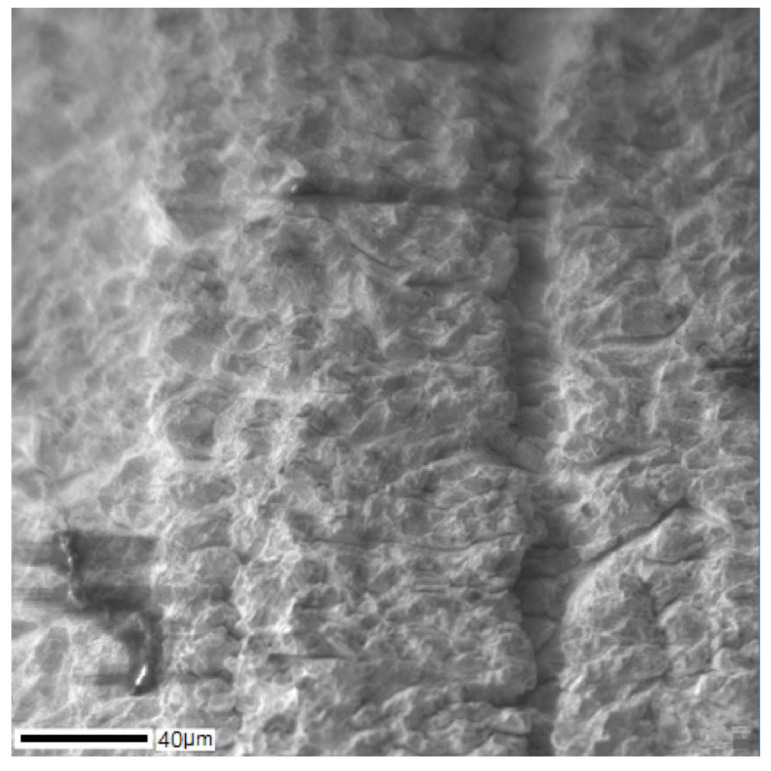
The surface of the Ti–28Nb–5Zr wire after drawing.

**Figure 11:**
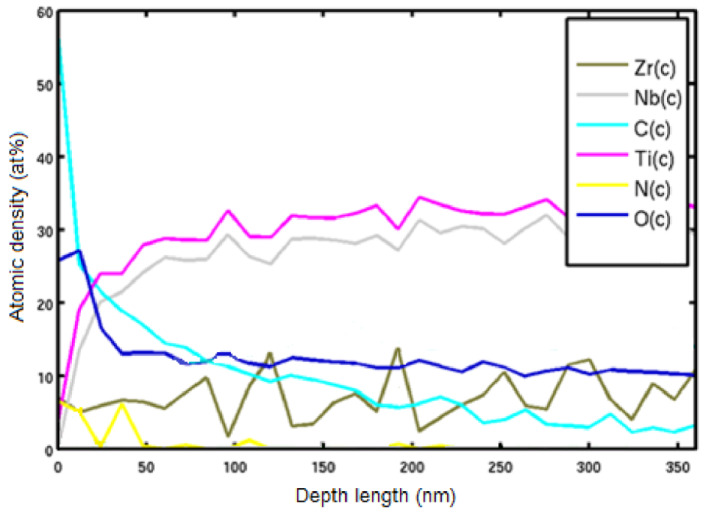
The surface composition of the polished wire of Ti–28Nb–5Zr alloy.

The surface morphology of the Ti–28Nb–5Zr alloy wire is shown in Figure 10. High roughness and heterogeneity were noted. However, in terms of chemical composition to a depth of more than 200 nm, a uniform oxide film without impurities was observed. After surface treatment, microdefects were smoothed out and the oxide layer decreased to 50 nm (Figure 11).

**Figure 12:**
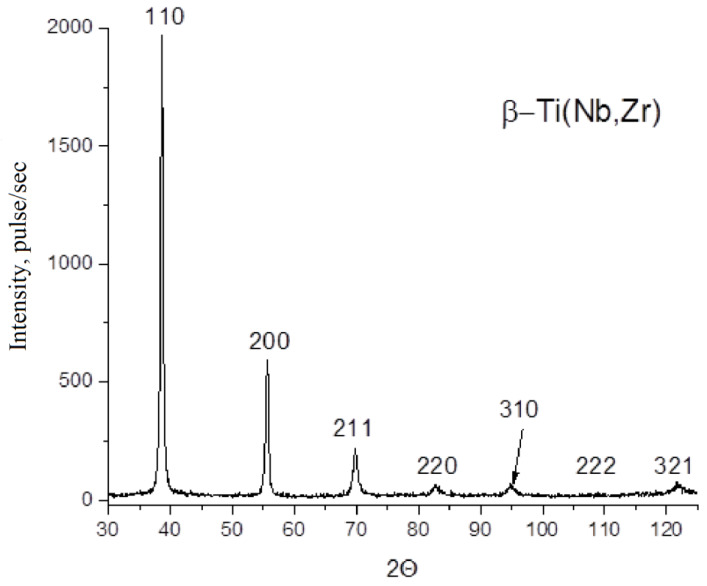
X-ray diffraction (XRD) patterns of Ti–28Nb–5Zr wire after drawing.

All wire samples were single-phase with a β-Ti (bcc) type crystal lattice (Figure 12). All samples were polycrystalline, without a pronounced texture, with broadened reflections without splitting the doublet even at large angles, which indicates a high degree of internal microstresses.

**Figure 13:**
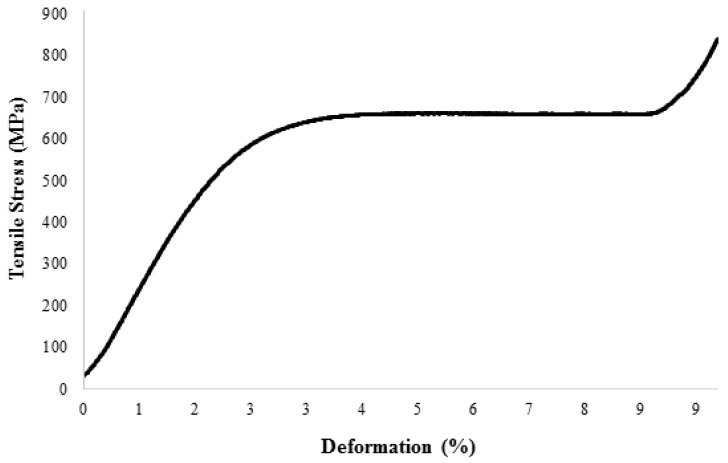
Results of uniaxial tensile tests of Ti–28Nb–5Zr wire after annealing at 800 °C for 1 h in vacuum.

The results of the mechanical testing are shown in Table 1 and Figure 13. The proof strength, plastic extension Rp0.2 of the samples after drawing was on average 580 MPa, tensile strength—705 MPa, Young’s modulus—38 GPa and elongation was of about 2%. Carrying out stabilizing annealing at 500 °C contributed to a significant increase in plasticity and a decrease in strength and Young’s modulus. The latter uniformly decreased with increasing annealing temperature. The proof strength, plastic extension Rp0.2 and tensile strength increased with increasing annealing temperature. Plasticity decreased at a temperature of 600 °C but reached high values at 700–800 °C.

**Figure 14:**
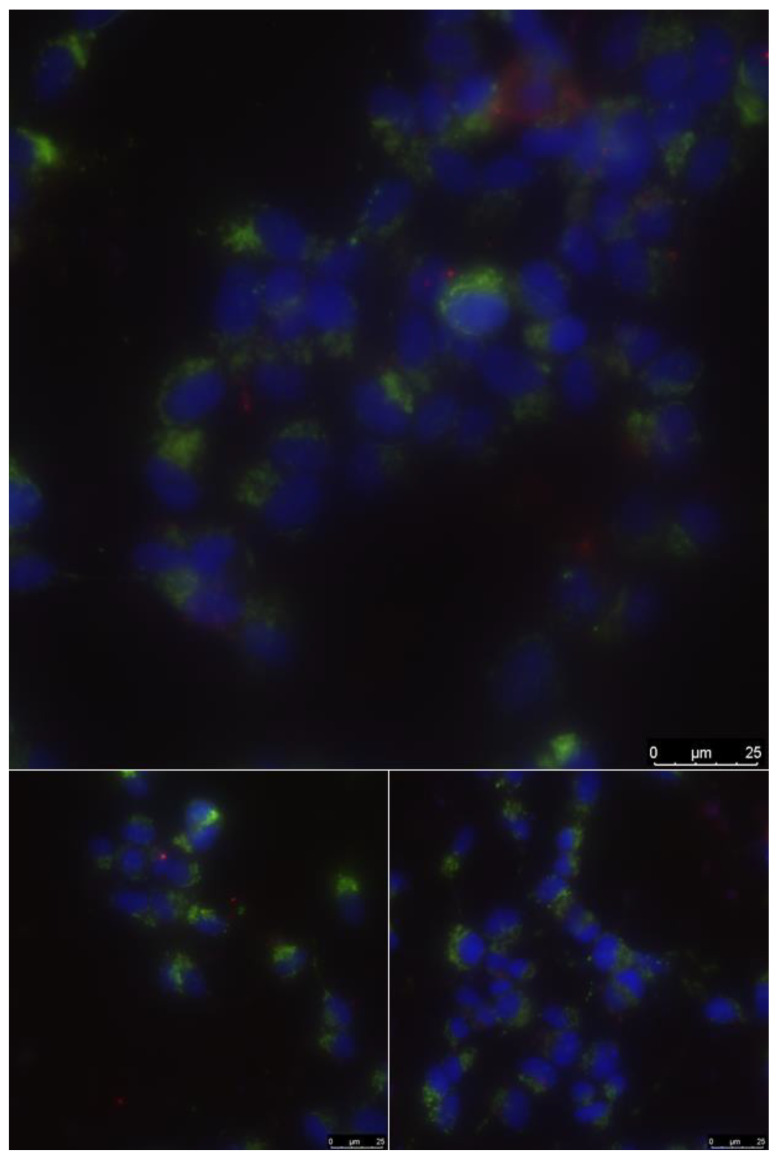
Cell culture on a Ti–Nb–Zr alloy sample. Channel overlay (Hoe fluorescence—blue nuclei of living cells, propidium iodide (PI) fluorescence—red dead cells, fluorescence MitoTracker Deep Red (MTDR)—green cell mitochondria). Several random sections of the sample.

Data on cell density, mitotic index, and the number of non-viable cells in the presence of the wire obtained are presented in Table 2. Figure 14 shows the results of fluorescence microscopy in several sections of the sample. The number of cells per area of 1 mm^2^ (by the number of nuclei stained with Hoechst 33342, which penetrates the plasma membrane and binds to DNA in the nucleus), the percentage of cells in the state of division (mitotic index, MI, is determined by the presence of visible chromosomes), the number of dead cells (according to the fluorescence of propidium iodide, which does not penetrate viable cells, but stains DNA in the nuclei of dead cells and cells with impaired membrane permeability) were estimated. All samples were biocompatible. Mitochondrial activity and good cell survival were observed.

## 4. Discussion

Alloys with the shape memory effect and superelasticity have large-scale prospects of application in various fields of science and technology [31,32,33], especially in medicine when using only non-toxic elements [34]. In particular, it is required for the production of endoscopic minimally invasive medical devices such as “stent” and “cava-filter” of a new generation. The most responsible and controversial step in producing such an alloy is its production in the form of a wire: material embrittlement processes are possible with undesirable oxygen accumulation within grain boundaries.

In the course of the work, complex studies of the structure and properties of the Ti–Nb–Zr alloy in the form of samples of various configurations from ingots to wire with a diameter of 1200 μm containing non-toxic elements and satisfying the implant’s requirements for mechanical, corrosion and biocompatible properties were carried out.

A homogeneous structure was noted at all stages. Large dendrites observed in ingots after smelting (Figure 1 and Figure 2) are consistent with the previous study, where the structure of Ti–Nb–Zr alloys had zones along the cross-section of the ingot from dendrites to large grains [35].

Dendrites are complex crystalline formations of a tree-like branching structure, which are the initial stage of crystal formation [36]. A crystal begins to form from the center of crystallization. In this case, dense packing of crystalline groups into one crystal does not work out; first, these groups are connected in certain directions, forming the axis of the future crystal. If the crystallization conditions are such that the spaces between the axes do not have time or cannot fill, the dendrite form is preserved and can be observed. The dendritic structure of the smelted ingot is defective. It is a chemical segregation. To eliminate the dendritic structure, long-term homogenizing annealing is used to activate the diffusion and alignment of the chemical composition.

The temperature range of homogenizing annealing was chosen, assuming that at 900 °С the processes of dendrite dissolution and recrystallization will begin, and after annealing at a temperature of 1000 °С a completely recrystallized β-phase will form [20,28,35]. It was noted that higher-temperature annealing leads to a sharp increase in grains to millimeter sizes, the fragility of the ingot, which will negatively affect the deformability of the ingots during further rolling.

The difference in mechanical properties between alloys with different grain sizes can be explained by greater or lesser development of the boundaries between the grains. These boundaries are grain separation surfaces in which the atoms of the metal itself are already energetically different from the atoms located in the lattice inside the grain. Thus, between the grains, there is a layer (film) in the form of indefinitely arranged atoms, which can affect the properties of the whole metal (alloy). For example, if these films are fragile, the bond between the grains will be weakened, and the destruction of the metal under mechanical action will occur along the grain boundaries. In this case, an intercrystalline fracture of the metal is observed. In the case when the layers between the grains are stronger than the grains themselves, the destruction will occur inside the grains. In such cases, an intracrystalline fracture of the metal is observed.

The fine-grained structure is characterized by higher mechanical properties. This can be explained by the large development of grain boundaries. The grain boundaries are obstacles to the development and movement of dislocations. In small grains, they will be shorter. Accordingly, in the case of an intracrystalline fracture of the metal, it is less likely that, as a result of the slip of the dislocations, they will assemble into a large defect, which will serve as the source of the crack. In the case of an intercrystalline fracture of a metal, it will be much more difficult for a crack to move along the developed, disoriented boundaries of a fine-crystalline structure than along the long boundaries of a coarse-grained structure.

The selected annealing mode led to the uniform dissolution of dendrites (Figure 3 and Figure 4), according to [35] contributing to the production of grains 200–400 microns in size, which is quite suitable for further plastic deformation of the melted ingots.

During rolling, the grains of the alloy decreased in size (Figure 5 and Figure 6), which indicates the absence of recrystallization at 600 °C during pre-heating of the bar before forging in alloys of all compositions [28].

Plastic deformation by drawing took place with heating and annealing to 600 °C in air, but the grain boundaries were not etched during microstructural analysis (Figure 7), which indicates the absence of recrystallization. Thus, as a result of plastic deformation, a wire with a nanostructure is possibly obtained.

X-ray diffraction studies (Figure 12) confirm that the crystal structure responsible for the manifestation of superelasticity and shape memory effect is obtained—the Ti–28Nb–5Zr alloy has a pure β-phase, which confirms the assumption that Nb and Zr are effective β-phase stabilizing elements [37].

It is noticeable that annealing at 500 °C leads to an increase in plasticity, but a decrease in strength. It is assumed that the metal deformed during drawing substantially loses strength since the average dislocation density decreases significantly. With increasing annealing temperature, strength increases, however, the relative elongation is minimal for the samples annealed at 600 °C. It can be assumed that at this temperature, the metastable β-phase begins to decay non-uniformly in grain volume, which leads to a decrease in the plasticity characteristics. After a further increase in the annealing temperature, the metastable β-phase decomposes evenly (turning into stable beta or mixed beta + alpha), which increases the strength and plasticity characteristics [38,39]. In this work, the tensile strength of the alloy after annealing the wire at 800 °C turned out to be of the order of 840 MPa, the proof strength, plastic extension Rp0.2–635 MPa (Table 1), which is comparable or higher than the parameters for Ti–6Al–4V [40], Ti–Nb–Sn [31,41], Ti–25Ta [42] or Ti–Nb [43].

The alloy did not have a short-term toxic effect on cells growing on de novo surfaces (Figure 13, Table 2).

Thus, the structure and properties suggest that the alloy Ti–28Nb–5Zr, in particular in the form of a wire, is promising for use in biomedical purposes.

## 5. Conclusions

- An alloy of the composition Ti–28Nb–5Zr was obtained sequentially in the form of ingots, bars and wire by melting in vacuum electric arc furnaces, intermediate homogenizing annealing, and intensive plastic deformation.
- A uniform distribution of elements over the volume of ingots was noted, as well as a good agreement between the element concentrations obtained in the alloy and the expected calculated values corresponding to the selected weights of charge materials. It was found that a uniform structure was obtained before and after homogenizing annealing. Elements of the alloy are not distributed in it by individual fragments but are connected in a single structure. The ingots have an inherent dendritic structure characteristic for cast alloys.
- It was noted that after the plastic deformation of the ingots and annealing at 600 °C in air, the grain boundaries are not visible during microstructural analysis, which indicates the absence of recrystallization. The formation of a nanostructure is possible. The surface morphology of wires after drawing shows a high heterogeneity and roughness. However, in terms of chemical composition to a depth of more than 200 nm, a uniform oxide film without impurities is observed. After grinding the surface, its uniformity increases. All wire samples were single-phase with a β-Ti type crystal lattice (bcc).
- For the wire obtained after stabilizing annealing, the average proof strength, plastic extension Rp0.2 on the samples was 635 MPa, tensile strength was 840 MPa, Young’s modulus was 22 GPa, and elongation was 6.76%.
- No toxicity was detected.
- Thus, the use of TiNbZr wire in medicine, for example, for the production of implants, such as stents and cava filters, is promising.

## Figures and Tables

**Table 1:** Data on mechanical testing of wire depending on heat treatment.

Sample	A (%)	Rp0.2 (MPa)	Rm (MPa)	E (MPa)
After drawing	1.9 ± 0.1	581 ± 10	705 ± 10	0.38 × 10^5^
Annealing 500 °C, 1 h, vacuum	6.1 ± 0.1	447 ± 10	596 ± 10	0.35 × 10^5^
Annealing 600 °C, 1 h, vacuum	4.7 ± 0.1	461 ± 10	613 ± 10	0.38 × 10^5^
Annealing 700 °C, 1 h, vacuum	6.6 ± 0.1	494 ± 10	667 ± 10	0.29 × 10^5^
Annealing 800 °C, 1 h, vacuum	6.8 ± 0.1	635 ± 10	840 ± 10	0.22 × 10^5^

**Table 2:** Cell activity.

Сomposition	Сells/mm^2^	Dead Cells, %	MI, %	N *
Ti–28Nb–5Zr	1230	2.4	1.4	374

* N—total number of counted cells for a given sample.
